# Post-Translational Modifications of Proteins in Cytosolic Nucleic Acid Sensing Signaling Pathways

**DOI:** 10.3389/fimmu.2022.898724

**Published:** 2022-06-20

**Authors:** Yu Deng, Ying Wang, Lupeng Li, Edward A. Miao, Pengda Liu

**Affiliations:** ^1^ Lineberger Comprehensive Cancer Center, The University of North Carolina at Chapel Hill, Chapel Hill, NC, United States; ^2^ Department of Biochemistry and Biophysics, The University of North Carolina at Chapel Hill, Chapel Hill, NC, United States; ^3^ Curriculum in Genetics and Molecular Biology, The University of North Carolina at Chapel Hill, Chapel Hill, NC, United States; ^4^ Department of Immunology and Department of Molecular Genetics and Microbiology, Duke University, Durham, NC, United States; ^5^ Department of Microbiology and Immunology, The University of North Carolina at Chapel Hill, Chapel Hill, NC, United States

**Keywords:** post-translational modifications, DNA sensing, RNA sensing, innate immunity, enzymes

## Abstract

The innate immune response is the first-line host defense against pathogens. Cytosolic nucleic acids, including both DNA and RNA, represent a special type of danger signal to initiate an innate immune response. Activation of cytosolic nucleic acid sensors is tightly controlled in order to achieve the high sensitivity needed to combat infection while simultaneously preventing false activation that leads to pathologic inflammatory diseases. In this review, we focus on post-translational modifications of key cytosolic nucleic acid sensors that can reversibly or irreversibly control these sensor functions. We will describe phosphorylation, ubiquitination, SUMOylation, neddylation, acetylation, methylation, succinylation, glutamylation, amidation, palmitoylation, and oxidation modifications events (including modified residues, modifying enzymes, and modification function). Together, these post-translational regulatory modifications on key cytosolic DNA/RNA sensing pathway members reveal a complicated yet elegantly controlled multilayer regulator network to govern innate immune activation.

## Introduction

All cells express a selected subset of innate immune sensors to defend against pathogens. Activation of the innate immune response promotes the production of interferons and proinflammatory cytokines, triggers regulated cell death to clear intracellular pathogens, and promotes adaptive immune responses. Pathogen-associated molecular patterns (PAMPs), which are conserved pathogen-derived molecules (1–3), are recognized by germline-encoded pattern recognition receptors (PRRs) of the innate immune system. Distinct types of PRRs sense a variety of PAMPs. PRRs include Toll-like receptors (TLRs), Nod-like receptors (NLRs), retinoic acid-inducible gene I (RIG-I)-like receptors (RLRs), C-type lectin receptors (CLRs), the absent in melanoma 2 (AIM2)-like receptors (ALRs), and other nucleic acid sensors including cyclic GMP-AMP synthase (cGAS) (4, 5). PAMPs include lipopolysaccharides (LPS), flagellin, lipoteichoic acid, peptidoglycan, and nucleic acid acids. LPS is recognized by TLR4, peptidoglycan is sensed by TLR2 (6), flagellin is recognized by TLR5 and NAIPs, and dsRNA is detected by TLR3 (7). Activation of PRRs by their corresponding PAMPs induces innate immunity and inflammation to clear infection (2, 3, 8–10). This review only focuses on nucleic acid sensing PRRs.

Given that both invasive bacteria/viruses and host cells contain DNA and RNA, how to distinguish self from foreign nucleic acids at first glance seems challenging for host defense. Largely, this is achieved by at least three mechanisms including “availability,” “localization,” and “structure” (11). “Availability” refers to the local concentration, half-life, and whether the nucleic acid is covered by binding partners. “Localization” indicates various cellular compartments where nucleic acids can be detected including the plasma membrane, cytoplasm, and nucleus. “Structure” includes the nucleic acid sequence, secondary structures, and certain modifications occurring in these nucleic acids. Nucleic acid sensors will transduce signals to trigger innate immunity in facilitating transcription of interferon and regulated cell death. Dysregulation of nucleic acid sensing or signal transduction leads to susceptibility to infection and other human diseases, including autoimmune diseases, autoinflammation, and cancer (12). Thus, the activation of nucleic acid sensing is tightly controlled.

The control of nucleic acid sensing is achieved at multiple levels. Upon bacterial or viral infection, type I interferons are synthesized to function through either autocrine or paracrine signaling to activate various STAT pathways to boost transcription of interferon-stimulated gene (13). Host cells can increase the sensitivity of nucleic acid sensors by inactivating sensor inhibitors. Conversely, bacteria or viruses can disable these sensors *via* post-translational modifications or *via* inhibitory binding proteins. To date, a plethora of protein post-translational modifications have been reported including phosphorylation (on Ser, Thr, and Tyr residues), ubiquitination (on Lys residues), acetylation (on Lys residues), methylation (on Lys and Arg residues), hydroxylation (on Pro residues), oxidation (on Cys residues), SUMOylation (on Lys and Glu residues), glutamylation (on Glu residues), and amidation (on Gln and Asn residues). These protein modifications control protein localization, stability, activation, and function in a temporal and spatial manner either through direct allosteric conformational changes or through regulating protein binding partners (14). Here, we will summarize major post-translational modifications identified to date on key mammalian nucleic acid sensing pathways, hoping to provide an up-to-date review of the roles of these modifications in fine-tuning innate immune responses, as well as provide novel insights into potentials in targeting certain modifying enzymes in treating human diseases where innate immune sensing is dysregulated.

## Cytosolic DNA Sensing

Cytosolic DNA is a danger signal that can be derived from infectious bacteria or viruses. It can also arise from self-DNA, such as from damaged genomic DNA, mitochondrial DNA, or DNA released during apoptosis. Sensing of cytosolic DNA comprises an important component for mammalian innate immunity, and activation of the cytosolic DNA sensors leads to the production of type I IFNs, pro-inflammatory cytokines, and chemokines, as well as regulated cell death for antiviral/antibacterial responses. A large number of candidates have been proposed as cytosolic DNA sensors including members of the ALRs such as AIM2 (15–17), myeloid nuclear differentiation antigen (MNDA) (18), interferon-inducible protein X (IFIX) (19), and interferon-inducible protein 16 (IFI16) (20), as well as non-ALR sensors such as cGAS (21), meiotic recombination 11 homolog A (MRE11) (22), Ku heterodimers (Ku70/Ku80) (23, 24), LRR binding FLII interacting protein 1 (LRRFIP1) (25), DExD/H box helicases (DDX41) (26), Z-DNA binding protein 1 (ZBP1) (27), and RNA polymerase III (28, 29). Notably, activation of different cytosolic DNA sensors leads to distinct downstream signaling. For example, DNA binding and activation of AIM2 in macrophages trigger the formation of inflammasome complexes for caspase 1 activation, leading to pyroptosis (30). Meanwhile, activation of other cytosolic DNA sensors such as cGAS stimulates interferon production. Specifically, DNA binding promotes cGAS dimerization and phase transition to facilitate cGAS activation, which leads to the synthesis of 2′3′-cyclic-GMP-AMP (cGAMP). cGAMP is a second messenger that diffuses throughout the cytosol and binds to a stimulator of interferon genes (STING), an endoplasmic reticulum (ER) transmembrane protein. This dimerizes STING, which then recruits and activates TBK1 to phosphorylate IRF3, promoting IRF3 dimerization and nuclear translocation, thus inducing IRF3-mediated IFN transcription (31). In addition, AIM2 recognizes specific DNA sequences in a cell type-dependent manner (32, 33), while cGAS senses cytosolic DNA in a DNA sequence-independent but DNA length-dependent manner in most cell types. Regardless of different types of cytosolic DNA sensors, hyperactivation of cytosolic DNA sensing and signaling results in autoimmune disease (34), while suppression of cytosolic DNA sensing contributes to evasion of immune destruction during tumorigenesis as well as resistance to cancer immunotherapies (35). Thus, activation of the nucleic acid sensors is tightly controlled under physiological conditions, and dysregulation leads to human pathological conditions. Post-translational modifications occurring on nucleic acid sensing pathway members serve as a critical approach to control and fine-tune pathway activities.

## Post-Translational Modifications of Cytosolic DNA Sensing

### Phosphorylation

Protein phosphorylation has been widely observed in nature as a reversible modification occurring on Ser, Thr, or Tyr residues to acutely control protein function—a phosphate group is added to target proteins by protein kinases and removed by protein phosphatases (36). Phosphorylation has been observed to recruit binding partners such as well-defined BRCT domains as readers for pSQ/pTQ motifs in DNA damage response (37), regulate protein stability (38), change protein cellular localization (39), or allosterically regulate enzyme activities (40). In this section, we will summarize discoveries associated with phosphorylation-mediated regulations in cytosolic DNA sensing.

**Figure 1:**
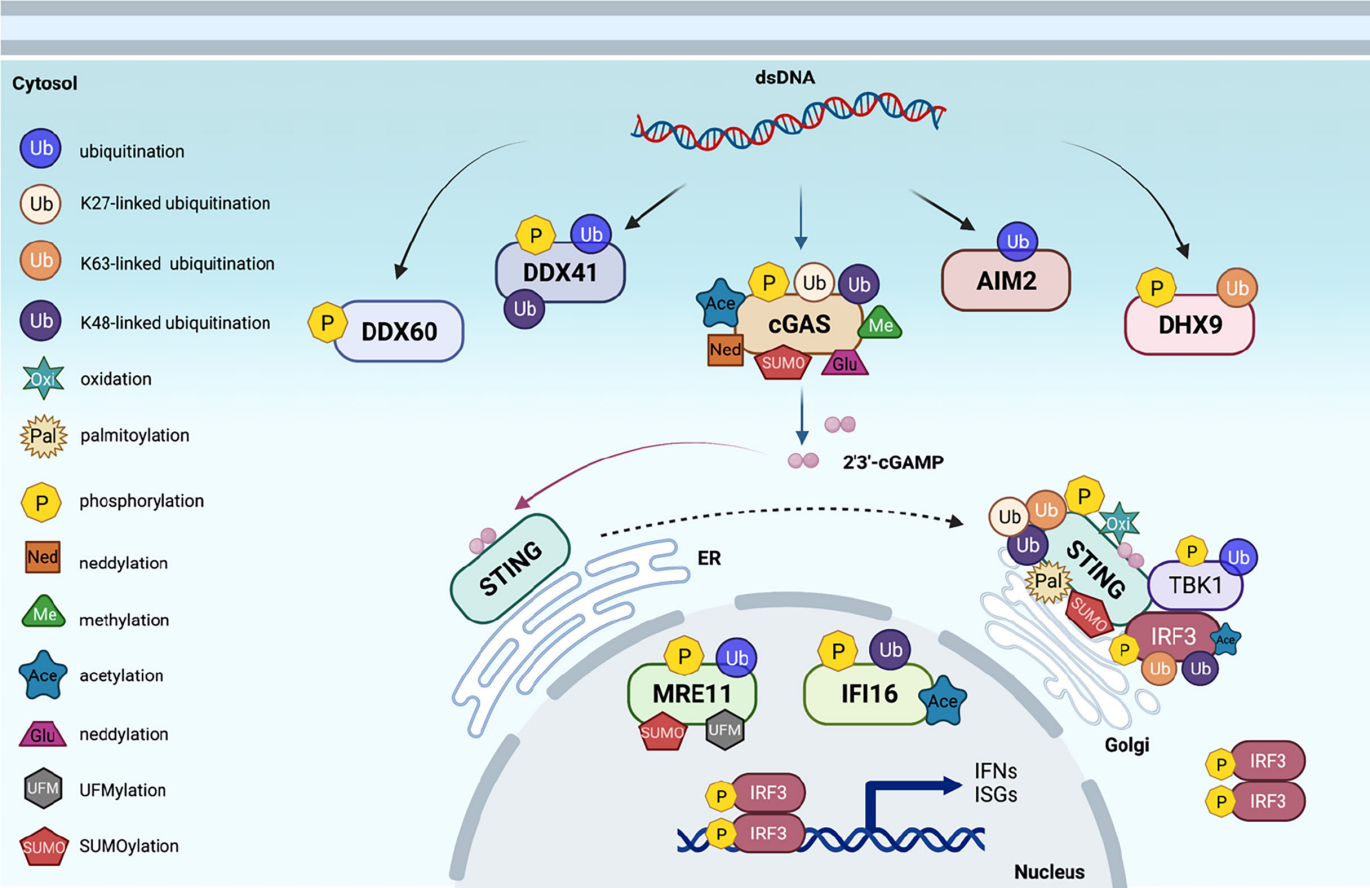
Post-translational modifications of proteins in cytosolic DNA sensing signaling. An overview of cytosolic DNA sensing signaling. Reported post-translational modifications on each DNA sensing signaling pathway member are earmarked by indicated icons. The cartoon illustration is generated by BioRender.

The cytosolic DNA sensor cGAS was reported to be phosphorylated on hS305 (human S305 equivalent to mS291: mouse cGAS-S291) by the kinase Akt confirmed by both *in vitro* kinase assays and mass spectrometry analyses (40) (**Table 1** and **Figure 1**). In addition, the same residue was also reported to be phosphorylated by CDK1 during mitosis, which was validated by both *in vitro* kinase assays and specific phospho-antibodies (41). Phosphorylation of cGAS on hS305/mS291 in the cGAS enzymatic domain by either kinase suppresses cGAS activity. Considering Akt activation is cell cycle dependent, peaking in S/G2 (101), it is plausible that Akt and CDK1 phosphorylate cGAS at S/G2 and M phases, respectively, to suppress cytosolic cGAS activation. Given that nuclear cGAS has been shown to be suppressed by binding to BAF (102) or tethering to chromatin (103), and during mitosis nuclear DNA is freely accessible to the cytosolic space, cGAS phosphorylation at its N-terminus by mitotic kinases including Aroura kinase B at multiple sites was observed to prevent cGAS sensing chromatin DNA, which tightly keeps cGAS inactive (44). Upon mitosis exit, the phosphatase PP1 dephosphorylates cGAS at hS305/mS291 to restore the ability of cytosolic cGAS in sensing DNA (41). Thus, cGAS phosphorylation in either its enzymatic domain or N-terminus may function in parallel to BAF1 (barrier-to-autointegration factor 1) binding or chromatin tethering in inhibiting cGAS activation during mitosis. Activation of cGAS in mitosis promotes mitotic cell death (104). Given cGAS largely senses cytosolic DNA, retention of cGAS in the cytoplasm at least through BLK (B lymphocyte kinase)-mediated cGAS-Tyr215 phosphorylation (42) facilitates its cytosolic DNA sensing and also evades its nuclear binding to PARP1 (Poly(ADP-Ribose) Polymerase 1) in suppressing homologous recombination. In addition, cGAS phosphorylation was also found to control cGAS activation by modulating cGAS oligomerization. Specifically, through a screen to search for compounds inhibiting VSV infection in THP1 cells *in vitro*, DNAPK (DNA-dependent protein kinase) inhibitors were found to restrict VSV replication by activating cGAS (43). Moreover, DNAPK was found to phosphorylate hcGAS on T68 and S213, which prevents cGAS oligomerization and activation. This study may provide explanations for why missense mutations of PRKDC, the DNAPK catalytic subunit, are observed in patients with autoimmune diseases (43). At resting states, cGAS is associated with the protein phosphatase PPP6C to retain cGAS in a dephosphorylated state, and upon DNA virus infection, dissociation of PPP6C allowed hcGAS phosphorylation on S435 (mcGAS-S420) residue in the catalytic pocket priming cGAS for activation (45). Thus, depending on the phosphorylation sites, cGAS phosphorylation can either suppress or facilitate cGAS activation.

**Table 1:** Post-translational modifications of proteins in cytosolic DNA sensing signaling pathways.

Protein	Post-translational modification	Modifying enzyme	Modification site(s)	Function	Reference
cGAS	Phosphorylation	Akt	mS291/hS305	Inhibits cGAS enzymatic activity	(40)
	Phosphorylation	CDK1	mS291/hS305	Inhibits cGAS enzymatic activity	(41)
	Phosphorylation	BLK	hY215	Facilitates cGAS cytosolic retention	(42)
	Phosphorylation	DNA-PK	hT68/hS213	Inhibits cGAS enzymatic activity	(43)
	Phosphorylation	Aurora kinase B	hS13/S37/S64/T69/T91/S116/S129/S143	inhibits cGAS activity during mitosis	(44)
	Dephosphorylation	PPI	mS291/hS305	Restores cGAS activity in the cytoplasm upon mitotic exit	(41)
	Dephosphorylation	PPP6C	mS420/hS435	Prevents cGAS from binding to GTP and inhibits cGAS activity	(45)
	Mono-ubiquitination	TRIM56	mK335	Promotes cGAS dimerization and DNA-binding	(46)
	Polyubiquitination	RNF185	mK173/mK384 (K27-linked)	cGAS activation	(47)
	Deubiquitination	USP14	hK414 (K48-linked)	cGAS stabilization	(48)
	Deubiquitination	USP27X	(K48-linked)	cGAS stabilization	(49)
	Deubiquitination	USP29	hK271 (K48-linked)	cGAS stabilization	(50)
	SUMOylation	TRIM38	mK217/mK464/hk231/hK497	cGAS stabilization	(51)
	DeSUMOylation	SENP2	mK217/mK464	cGAS stabilization	(51)
	DeSUMOylation	SENP7	mK335/mK372/mK382	cGAS activation by enhancing cGAS dimerization and DNA-binding	(52)
	Poly-neddylation	RNF111	hK231/hK421	cGAS dimerization and activation	(53)
	De-neddylation	SENP8	hK231/hK421	cGAS inhibition	(53)
	Methylation	PRMT5	hR124	cGAS inhibition by blocking DNA binding	(54)
	Acetylation	KAT5	hK47/hK56/hL62/hK83	facilitates DNA binding and cGAS activation	(55)
	Deacetylation	HDAC3	hK384/hK394/hK414	facilitates DNA binding and cGAS activation	(56)
	Poly-glutamylation	TTLL6	mE272/hE286	cGAS inhibition by blocking DNA binding	(57)
	Mono-glutamylation	TTLL4	mE302/hE314	cGAS inhibition	(57)
	Deglutamylation	CCP5	mE302	cGAS activation	(57)
	Deglutamylation	CCP6	mE272	cGAS activation	(57)
IFI16	Phosphorylation	pUL97		IFI16 relocalization to cytoplasm	(58)
	Poly-ubiquitination	TRIM21	hK3/K4/K6 (K48-linked)	IFI16 degradation	(59)
	Ubiquitination	ICP0		IFI16 degradation	(60)
	Acetylation	p300	within NLS	IFI16 cytoplasmic retention	(61)
AIM2	Deubiquitination	USP21		AIM2 stabilization	(62)
	Degradation by selective autophagy	TRIM11		AIM2 degradation *via* p62-dependent selective autophagy	(63)
DHX9	Phosphorylation	PI3KKs	S279/S321	Chemoresistance	(64)
	Ubiquitination	SPOP	(K48-linked)	DHX9 degradation	(65)
	Ubiquitination	RNF168	(K63-linked)	DHX9 recruitment to R-loop-prone genomic loci	(66)
DDX41	Phosphorylation	BTK kinase	hY414	DDX41 activation	(67)
	Ubiquitination	TRIM21	hK9/hK115	DDX41 degradation	(68)
DDX60	Phosphorylation	EGFR	hY793/hY796	Type 1 INF production	(69)
MRE11	Phosphorylation	CK2, PLK1	hS649/hS688	MRN complex assembly to initiate DNA repair	(70)
	Phosphorylation	ATM	hS646/hS678	The MRC complex disruption upon DNA damage	(71)
	Phosphorylation	PLK1	hS688	MMAP-MRN complex formation	(72, 73)
	Phosphorylation	RSK	hS676	Disrupts MRE11 binding to DNA	(74)
	Phosphorylation	P70-S6K		MRN complex disruption	(75)
	Ubiquitination	UBQLN4		MRE11 degradation	(76)
	Ubiquitination	clAP2		MRE11 degradation	(77)
	UFMylation		hK282	MRN complex recruitment to damaged DNA	(78)
	UFMylation		hK281/hK282	Maintaining telomere length and aiding cell survival	(79)
	Methylation	PRMT1	haa566-600	Intra-S-phase DNA damage checkpoint response	(80)
STING	Phosphorylation	TBK1	hS366/mS365	STING activation	(81)
	Phosphorylation	ULK1	hS366/mS365	STING degradation	(82)
	Phosphorylation	TBK1/ULK1/2	mS365	STING activation by facilitating recruitment of “Senp2”	(51)
	Ubiquitination	RNF5	hK150	STING degradation	(83)
	Ubiquitination	TRIM30a	hK275	STING degradation	(84)
	Ubiquitination	TRIM29	hK370	STING degradation	(85)
	Ubiquitination	RNF26	hK150	STING stabilization	(86)
	Ubiquitination	TOLLIP		STING stabilization at resting states	(87)
	Ubiquitination	RNF115	hK20/K224/K289	STING activation and TBK1 recruitment	(88)
	Ubiquitination	TRIM56	hK150	STING dimerization and activation	(89)
	Ubiquitination	AMFR	hK137/hK150/hK224/hK236	STING activation and TBK1 recruitment	(90)
	Ubiquitination	TRIM32	hK20/K224/K236	STING activation and TBK1 recruitment	(91)
	Ubiquitination	MUL1	hK224	STING trafficking and activation	(92)
	Deubiquitination	USP20	(K48-linked)	STING stabilization	(93)
	Deubiquitination	EIF3S5	(K48-linked)	STING stabilization	(94)
	Deubiquitination	CYLD		STING stabilization	(95)
	Deubiquitination	USP13	haa301-863 (K63-linked)	Impairs STING binding to TBK1	(96)
	Deubiquitination	MYSM1	hK150 (K63-linked)	STING inhibition	(97)
	Deubiquitination	USP21		STING inactivation	(98)
	SUMOylation	TRIM38	hK338	STING stabilization and activation	(51)
	De-SUMOylation	SENP2		STING degradation	(51)
	palmitoylation	DHHC3/DHHC7/DHHC15	hC88/C91	STING trafficking and activation	(99)
	Oxidation		hC148/mC147	STING inactivation	(100)

The orange color indicates activation of the indicated molecule by indicated modifications; the blue color indicates suppression of the indicated molecules by indicated modifications.

Human cytomegalovirus (HCMV) infection on human embryonic lung fibroblasts induced viral pUL97-mediated phosphorylation on IFNγ-inducible protein 16 (IFI16), which facilitates the mis-localization of IFI16 into the cytosol to disable its viral DNA sensing ability (58) (**Table 1**). DHX9 phosphorylation close to its substrate-binding domain (may include S239 and S321) by PI3KKs promoted oncogenic circular RNA expression contributing to chemoresistance (64). BTK-mediated DDX41 phosphorylation on Tyr414 was critical for sensing foreign dsDNA and subsequent recruitment of STING for IFN production (67). However, the underlying mechanism(s) for how these phosphorylation events control the function of these sensors remains unclear.

Plk1 phosphorylates MRE11 at S649 and S688 residues, and CK2 phosphorylates MRE11 at S688, both of which promote the assembly of the MRB complex that is necessary to imitate the DNA damage repair (70). In addition, Plk1-mediated MRE11 phosphorylation at S688 also promotes MRN binding to MMAP (C2orf44) to form the MMAP-MRN complex, which further facilitates the repair of damaged DNA (72, 73). In contrast, RSK-mediated MRE11-S676 phosphorylation interferes with MRE11 binding to DNA, leading to impaired homologous recombination (74). Similarly, S6K phosphorylates MRE11-T597 residue, leading to impaired MRN complex formation and subsequent deficient DNA damage repair in colon cancer cells (75). Although it is clear that these various phosphorylation events exert distinct regulatory effects in modulating MRE11 function, whether these phosphorylation events are regulated under viral/bacterial infection during the cytosolic sensing process remains to be determined.

As an ER-localized protein, STING binds di-nucleic acids including 2′3′-cGAMP generated by cGAS upon sensing cytosolic DNA, which facilitates STING dimerization, oligomerization, and trafficking to Golgi, where TBK1 binds STING and phosphorylates STING-S366 (S365 in mice) (81), which is necessary to further recruit IRF3. TBK1 then also phosphorylates IRF3, promoting IRF3 dimerization and nuclear translocation to induce transcription of interferon genes. Afterward, 2′3′-cGAMP also triggers ULK1 activation by releasing its suppression by AMPK to phosphorylate STING-S366, leading to STING degradation, thereby preventing sustained innate immune signaling (82). One possible mechanism to explain how STING-S366 phosphorylation primes STING for degradation might be mediated by STING deSUMOylation, such that STING-S366 phosphorylation promotes SENP2 recognition at the late stage of viral infection that facilitates STING deSUMOylation, allowing STING ubiquitination to occur for STING degradation (51).

### Ubiquitination

Protein ubiquitination is an ancient and evolutionarily conserved protein modification in regulating protein function in eukaryotes (105). Ubiquitin is a protein with 76 amino acids containing seven lysine residues that can be conjugated with another ubiquitin molecule to form polyubiquitin chains with distinct lengths. Ubiquitin can also be conjugated in a head-to-toe manner so that overall there are 8 distinct ubiquitin linkages formed including linear (M1, head-to-toe), K6, K11, K27, K29, K33, K48, and K63. To date, K11- and K48-linked ubiquitination has been related to proteasomal protein degradation, and other linkages have been reported to be involved in other biological processes including DNA damage response, protein trafficking, structure, and activity control (106). The ubiquitination process is carried out by a three-step enzymatic cascade including activating ubiquitin by E1, conjugating ubiquitin by E2, and selection of specific substrates for ubiquitin modification by E3 ubiquitin ligases. Given that E3 determines the substrate specificity, there are more than 600 identified E3 ligases in mammals. The poly-ubiquitin chains can be removed by deubiquitinases (DUBs) and largely consist of USPs, OTUs, UCHs, Joshphines, MINDYs, and JAMMs families (107). These DUBs exert ubiquitin chain hydrolysis ability and recognize both ubiquitin chains and substrates. Thus, protein ubiquitination is a dynamic and reversible process governed by E1/E2/E3 and DUBs.

Ubiquitination has been extensively studied in regulating innate immune DNA sensing signaling. Mono-ubiquitination of mcGAS on K335 by the E3 ligase TRIM56 was reported to facilitate cGAS activation upon DNA challenge by enhancing DNA binding and cGAS dimerization (46) (**Table 1**). RNF185-mediated mcGAS poly-ubiquitination at K173 and K384 residues through a K27 linkage also propagate the cGAS enzymatic activity (47). In contrast, various DUBs have been reported to facilitate cGAS activation largely by stabilizing cGAS proteins—for example, USP14 removes K48-linked polyubiquitin chains on K414 (48), USP29 removes polyubiquitination on K271 (50), and USP27X cleaves K48-linked ubiquitin chains on cGAS (49) to antagonize cGAS degradation. Notably, the E3 ligases governing proteasomal cGAS ubiquitination and degradation remain unclear.

TIRM21-governed IFI16 polyubiquitination on K3/K4/K6 residues earmarks IFI16 for degradation (59). Interestingly, herpesviral nuclear protein ICP0 binds nuclear IFI16 to retain it in the nucleus and additionally facilitates its degradation (60), leading to evasion of innate immune surveillance. Upon DNA virus infection, the E3 ubiquitin ligase TRIM11 binds AIM2, enhancing TRIM11 association with p62 and leading to AIM2 degradation through selective autophagy (63). In contrast, USP21 deubiquitinates AIM2, stabilizing the AIM2 inflammasome and facilitating downstream inflammation signaling (62). The DNA helicase DHX9 has been reported to be ubiquitinated and degraded by the E3 ligase SPOP (65), while RNF168-mediated DHX9 ubiquitination promotes recruitment of DHX9 to genomic loci prone to form R-loops where DHX9 resolves and removes R-loops (66). Whether any of these ubiquitination events occur in cytosolic DNA sensing mediated by DHX9 remains to be further investigated.

The expression of the E3 ligase TRIM21 is induced by interferons, and TRIM21 promotes K48-linked DDX41-K9 and K115 ubiquitination and degradation, serving as a mechanism to restrain the activation of innate immunity upon cytosolic DNA challenges (68). Upon DNA damage, UBQLN4 is recruited to damaged DNA, where UBQLN4 binds ubiquitinated MRE11 to remove it from repairing damaged DNA, leading to degradation of MRE11 to terminate the homologous recombination (76). The E3 ligase cIAP2 binds MRE11 to downregulate MRE11 protein levels by inducing an altered ubiquitination pattern on MRE11 (77).

Ubiquitin modifications on STING have been extensively studied with distinct effects on STING function in innate immunity. The E3 ligase RNF5 has been reported to target STING-K150 for ubiquitination and degradation upon viral infection (83), a process that can be antagonized by RNF26-mediated STING-K150 ubiquitination, presumably through a non-K48 linkage (86). In addition, TRIM29 (85) and TRIM30a (84) have also been reported to ubiquitinate STING-K370 and K275 residues, respectively, to target STING for degradation, serving as mechanisms to restrain innate immune sensing. As a result, *Trim30a*-deficient mice are more resistant to DNA viral infection (84). Interestingly, TRIM30a expression is induced by HSV-1 infection, suggesting that TRIM30a-mediated STING ubiquitination and degradation may serve as a negative feedback mechanism to shut down interferon signaling to avoid its hyperactivation (84). Moreover, TOLLIP, which usually helps to clear poly-Q-containing protein aggregates, was found in a siRNA-mediated screen as a positive regulator to stabilize STING proteins at the resting state by binding STING to prevent its lysosomal degradation (87). Other than regulating STING protein stability, STING ubiquitination by various E3 ligases has also been shown to be critical for STING dimerization/oligomerization and recruitment of both TBK1 and IRF3. For example, RNF115-mediated STING-K20/K224/K289 ubiquitination facilitates the formation of higher orders of STING structures and TBK1 recruitment (88). TRIM56-dependent STING-K150 (89), AMFR, governed STING-K137/K150/K224/K236 (90), and TRIM32-mediated STING-K20/K224/K236 ubiquitination (91) plays critical roles in STING dimerization and recruitment of TBK1/IRF3 to facilitate IRF3 phosphorylation and interferon production presumably through non-K48-linked ubiquitin chain linkages. In addition, the E3 ligase MUL1 conjugates K63-linked ubiquitin chains to STING-K224, which facilitates proper STING trafficking from ER to Golgi and bridges interactions of TBK1 with IRF3 mediated by STING (92).

DUBs have also been identified to antagonize E3 ligase-induced STING ubiquitination and function. Three DUBs including USP20 (93), EIF30S (94), and CYLD (95), have been reported to largely remove K48-linked polyubiquitin chains on STING, leading to stabilization of STING proteins and sustaining innate immune signaling. Another two DUBs, including USP13 (96) and MYSM1 (97), largely cleave K63-linked ubiquitin chains from STING, leading to impaired STING recruitment of TBK1 and IRF3, resulting in dampened interferon production. USP21 also negatively regulates STING function in promoting interferon production by deubiquitinating STING (98), a process negatively controlled by p38-MAPK (98).

### SUMOylation and Neddylation

In addition to ubiquitin, other ubiquitin-like molecules can also be conjugated to target proteins to modulate their function. This includes SUMO (small ubiquitin-related modifier), NEDD8 (neural precursor cell expressed developmentally downregulated protein 8), and UFM1 (ubiquitin-fold modifier 1, ISG15 (ISG15 Ubiquitin-Like Modifier). SUMO is a ~10 KD small protein structurally similar to ubiquitin and can be conjugated to target proteins through lysine residues by an enzyme cascade consisting of E1-activating enzyme, E2-conjugating enzyme, and E3 SUMO ligase (108). SUMOylation regulates target protein stability, cellular location, and function largely through recruiting distinct subsets of downstream binding partners and effectors. NEDD8 is also a ubiquitin-like protein with NEDD8-specific conjugation and deconjugation pathways that can distinguish this modification from other ubiquitin-like modifications (109). The best-characterized proteins regulated by Nedd8 conjugation are cullins, which are scaffold proteins for cullin-ring types of E3 ubiquitin ligases (110), and there are also non-cullin protein targets found with neddylation (109). The UFM1 system is less understood, although it is highly conserved in eukaryotes except for yeast and fungi. Different from other ubiquitin-like molecules, UFM1 is more connected with the function of ER and ER stress controls the UFM1 system (111). Modification of DNA sensing signaling components by SUMOylation, neddylation, or UFMylation has also been observed in controlling innate immune activity.

mcGAS was found to be SUMOylated by TRIM38 at the resting state on K271/K464, which antagonizes ubiquitination-mediated degradation, resulting in cGAS stabilization for acute sensing viral infection (51). At the later stage of infection, the deSUMOylase SENP2 cleaves SUMO conjugates added on cGAS-K217/K464 by TRIM38 to facilitate cGAS ubiquitination and degradation, thus restraining cGAS overactivation (51), while another deSUMOylase, SENP7, through removing SUMO conjugates on mcGAS-K335/K372/K382, facilitates cGAS binding to DNA and cGAS dimer formation and subsequent interferon production (52). Given that TRIM56 mono-ubiquitinates mcGAS on K335, it is plausible that SENP7-mediated cGAS deSUMOylation is necessary for cGAS mono-ubiquitination in order to activate cGAS (46). Similarly, TRIM38 also maintains STING SUMOylation on K338, which prepares STING ready for sensing cytosolic DNA signaling. Upon viral infection, SENP2 deSUMOylates STING, which facilitates STING degradation in terminating this signaling (51). Thus, TRIM38/SENP2 controls both cGAS and STING protein stability in the early and late stages of viral infection to ensure the timely activation and inactivation to fine-tune the pathway responses.

cGAS was also found to be neddylated by UBE2M (E2)/RNF111 (E3) on K231/K421 residues, where neddylated cGAS will be properly positioned to form dimers with the previous cGAS, thus facilitating cGAS activation. In contrast, SENP8 de-neddylates cGAS on these residues and subsequently impairs proper cGAS dimer formation and activation (53).

UFMylation of MRE11-K282 has been observed and reported to be critical for MRN complex formation to ensure a timely location of the MRN complex to damaged DNA (78). In addition, MRE11 UFMylation is also important to recruit the phosphatase PP1 to dephosphorylate NBS1, therefore enhancing MRN complex binding to telomeres to maintain telomere length (79).

### Other Protein Modifications

In addition to phosphorylation, ubiquitination, and ubiquitin-like modifications, other post-translational modifications that control activation of cytosolic DNA sensing have also been reported albeit with less attention. cGAS acetylation has been shown to be critical for cGAS binding to DNA. cGAS acetylation on K47/K56/K62/K83 residues in cGAS N-terminus by KAT5 facilitates DNA binding and cGAS activation (55), while deacetylation of cGAS-K384/K394/K414 in the cGAS enzymatic domain by HDAC3 is necessary for cGAS binding DNA (56). This may suggest that although both cGAS N and C domains participate in DNA binding, acetylation is only preferred in the disordered N but not well-ordered C domain for DNA recognition. PRMT5-mediated cGAS-R124 methylation attenuates cGAS-controlled antiviral immune response, largely through disrupting cGAS binding to DNA (54). Considering the R124 residue is also within the N-terminus, it is plausible that R124 methylation destabilizes cGAS conformation, while K47/K56/K62/K83 maintains a suitable cGAS structure for DNA binding, which requires further in-depth investigations. PRMT1 methylates MRE11 (aa566-600) to maintain an intact MRN complex during intra-S-phase DNA damage, which is critical to establish a proper intra-S-phase DNA damage checkpoint (80). In addition, P300 acetylates IFI16 within its nuclear localization signal (NLS) to retain IFI16 in the cytoplasm, disabling its ability to sense nuclear DNA for activation of the innate immune signaling (61).

In addition to S/T targeted phosphorylation and K/R targeted modifications, E (Glu) residues in cGAS have been observed to undergo glutamylation modifications. Specifically, TTLL4-mediated mono-glutamylation of hcGAS-E314 inhibits cGAS enzymatic activity (57), and similarly, TTLL6-governed poly-glutamylation of hcGAS-E272 disrupts cGAS binding to DNA (57). In contrast, CCP5 removes hcGAS-E314 mono-glutamylation, and CCP6 cleaves hcGAS-E272 poly-glutamylation to recover cGAS binding to DNA and activation (57). Both glutamylated and non-glutamylated cGAS species are observed at resting states, while during viral infection, expression of TTLL enzymes is downregulated, leading to increased populations of non-glutamylated cGAS for sensing viral DNA to initiate innate immunity.

Given that the cellular trafficking of STING from ER to Golgi plays a critical role in recruiting TBK1/IRF3 to activate the innate immunity, other modifications on STING than K63-linked ubiquitination (92) have also been observed. C88/C91 palmitoylation of STING by DHHC3/DHHC7/DHHC15 was reported to be necessary to mediate STING leaving ER for activation (99), while the detailed molecular mechanisms remain unclear. In addition to palmitoylation, cysteine residues also undergo oxidation, such that hSTING-C148 oxidation induced by cellular ROS interferes with STING oligomerization and subsequent activation to suppress interferon production (100).

## Overview of Cytosolic RNA Sensing

Similar to DNA sensing, infection by RNA viruses that expose viral RNA to host cytoplasm triggers acute nucleic acid sensing *via* PRRs to initiate signaling pathways leading to the production of type I interferons including IFNα and IFNβ and other cytokines for robust innate immune responses. Depending on the localization, RNA sensors can be divided into endosomal membrane-associated TLRs that function primarily in immune cells, and cytosolic RNA sensors RIG-I (112) and MDA5 (melanoma differentiation-associated gene 5), which are expressed in most cells (2). Both RIG-I and MDA5 are RNA helicases composed of two N-terminal CARDs (caspase recruitment domains), a central DExD/H-box ATPase/helicase domain and a C-terminal regulatory domain that binds RNA (112). RIG-I senses dsRNA, single-strand RNA with 5′-triphosphates (113), or even reversely transcribed 5′-triphosphate RNA from cytosolic viral dsDNA (29). In contrast, MDA5 largely recognizes dsRNA (114). RNA binding stimulates helicase activity in both RIG-I and MDA5 and promotes the formation of prion-like aggregates through oligomerization to expose N-terminal CARDs. These exposed CARDs bind the mitochondrial protein MAVS (also named VISA, IPS-1, CARDIF) (115) to form a signaling platform with the help of the E3 ligase TRAF3 (116) (also other TRAFs (117)) to recruit TBK1 and IKKε to facilitate transcription of interferon genes through IRFs (118) and activate IKKα/β to induce NF-κB-mediated transcription of proinflammatory genes (119). Phosphorylated IRF3 or IRF7 forms homo-dimers and translocates into the nucleus to promote type I interferon transcription.

## Post-Translational Modifications of Proteins in Cytosolic RNA Sensing

### Phosphorylation

**Figure 2:**
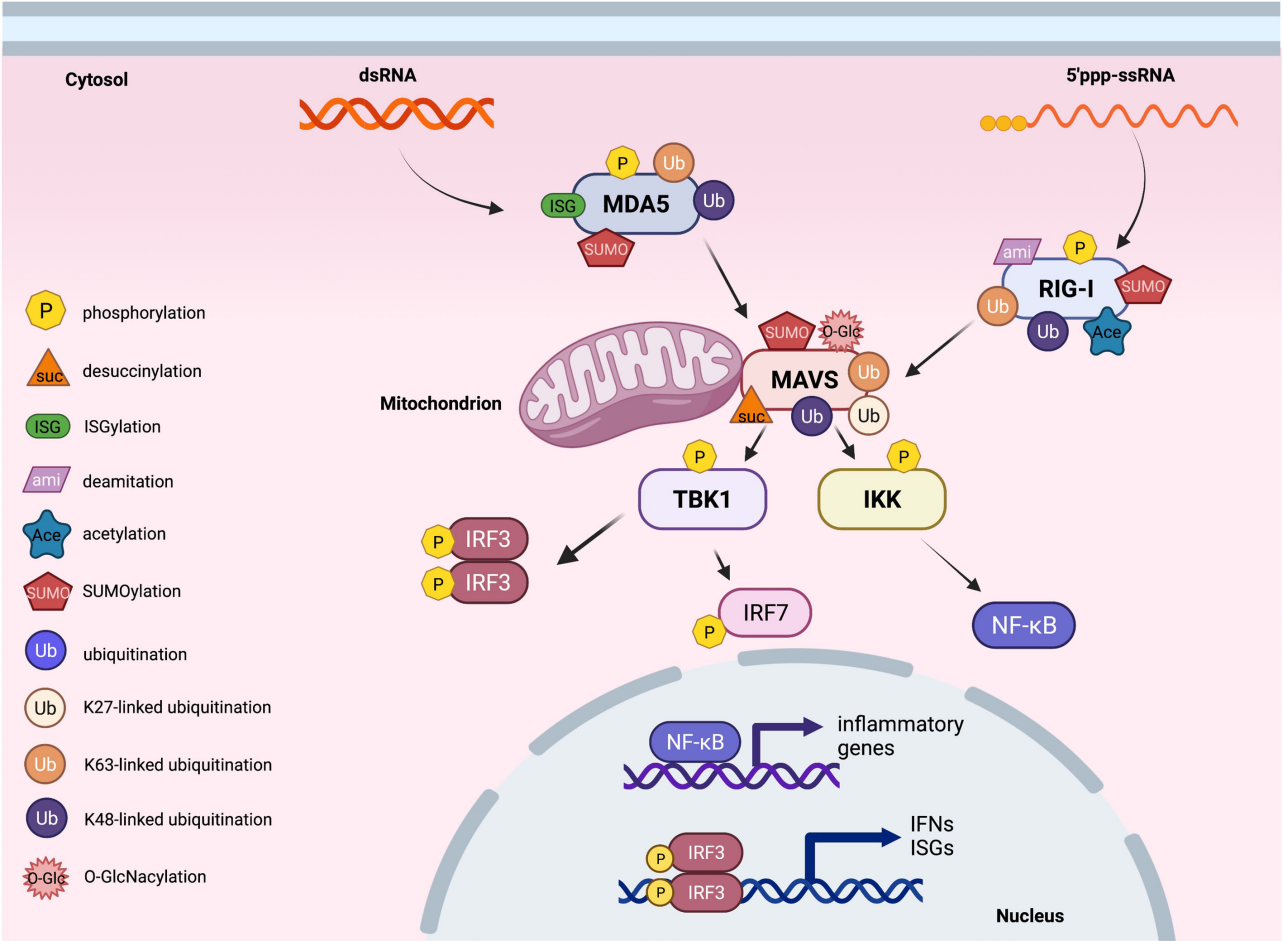
Post-translational modifications of proteins in cytosolic RNA sensing signaling. An overview of cytosolic RNA sensing signaling. Reported post-translational modifications on each RNA sensing signaling pathway member are earmarked by indicated icons. The cartoon illustration is generated by BioRender.

At resting states, both RIG-I and MDA5 are phosphorylated at caspase recruitment domains to keep them inactive. Upon sensing cytosolic RNA, TRIM25 adds K63-linked polyubiquitin on RIG-I at K172 to facilitate RIG-I binding with its downstream effectors MAVS (also known as VISA/IPS-1) (120), thus activating the RNA sensing signaling to induce interferon production (**Table 2** and **Figure 2**). Phosphorylation of RIG-I on S8 and/or T170 impairs its K172 polyubiquitination by PKCα/β *via* disrupting TRIM25 binding and also disrupts RIG-I interactions with MAVS and subsequent antiviral interferon production (137, 139). In addition, RIG-I phosphorylation by CKII at T770/S854/S855 inhibits RIG-I activation by inhibiting the formation of RIG-I intermolecular interactions and oligomerization (140). Similarly, MDA5 phosphorylation on S88 blocks MDA5 interaction with MAVS at resting states (141). In contrast, an RNAi screen identified PP1α and PP1γ as major phosphatases dephosphorylating RIG-I on S8/T170, which facilitates RIG-I binding to TIRM25 and MAVS (VISA/IPS-1) to promote innate immune activation (141). Therefore, PP1-depleted cells showed a decreased ability to induce interferon, and increased RNA virus replication upon RNA virus infection including influenza virus, paramyxovirus, dengue virus, and picornavirus (141). RNA viruses induced EGFR activation, which led to DDX60-Tyr793/Tyr796 phosphorylation, which attenuated RIG-I signaling and reduced type I IFN production (69).

**Table 2:** Post-translational modifications of proteins in cytosolic RNA sensing signaling pathways.

Protein	Post-translational modification	Modifying enzyme	Modification site(s)	Function	Reference
RIG-I	Ubiquitination		Unanchored chains (K63-linked)	RIG-I activation by binding RIG-I CARD domains	(121)
	Ubiquitination	TRIM25/EFP	hK172	RIG-I activation	(122)
	Ubiquitination	Riplet	(K63-linked)	RIG-I activation	(123)
	Ubiquitination	RNF 135	hK849/851 (K63-linked)	RIG-I activation	(124)
	Ubiquitination	REUL	hK154/164/172 (K63-linked)	RIG-I activation	(125)
	Ubiquitination	TRIM4	hK154/164/172 (K63-linked)	RIG-I activation	(126)
	Ubiquitination	MEX3C	hK99/169 (K63-linked)	RIG-I activation	(127)
	Ubiquitination	RNF122	hK115/146 (K48-linked)	RIG-I degradation	(128)
	Ubiquitination	RNF125	(K48-linked)	RIG-I degradation	(129)
	Ubiquitination	HOIL-1L/HOIP LUBAC		TRIM25 degradation and RIG-I K63 ubiquitination inhibition	(130)
	Ubiquitination	c-Cbl	hK813 (K48-linked)	Siglec-G induced by RNA viral infection facilitates SHP2 and c-Cbl binding and degradation of RIG-I	(131)
	Deubiquitination	CYLD	(K63-linked)	RIG-I inhibition	(132)
	Deubiquitination	USP3	(K63-linked)	RIG-I inhibition (K63-linked ubiquitin chain removal upon viral infection)	(133)
	Deubiquitination	USP21	(K63-linked)	RIG-I inhibition (K63-linked ubiquitin chain removal)	(134)
	Deubiquitination	USP4	(K48-linked)	Facilitates RIG-I activation by removing K48-linked ubiquitination	(135)
	Deubiquitination	USP15		Deubiquitylates and stabilizes TRIM25 to enhance TRIM25-mediated RIG-I ubiquitination and activation	(136)
	Phosphorylation		hT170	RIG-I inhibition *via* inhibiting K172 polyubiquitination	(137)
	Phosphorylation		hS8	RIG-I inhibition by inhibiting TRIM25 induced RIG-I ubiquitination	(138)
	Phosphorylation	PKC-α/β	hS8 and T170	RIG-I inhibition by inhibiting RIG-I binding with TRIM25 and MAVS	(139)
	Phosphorylation	CKII	hT770/S854/S855	RIG-I inhibition by inhibiting RIG-I multimerization	(140)
	Dephosphorylation	PP1α and PP1γ	hS8 and T170	RIG-I activation	(141, 142)
	SUMO	TRIM38	hK96/K888	RIG-I activation	(143)
	SUMO	SENP2		RIG-I inhibition	(143)
	Deamidation	PFAS	hQ10/N245/N445	RIG-I activation	(144)
	Deacetylation	HDAC6	hK909	RIG-I oligomerization and activation	(145, 146)
MDA5	Ubiquitination	RNF125	(K48-linked)	MDA5 degradation	(129)
	Ubiquitination	TRIM13	(K48-linked)	MDA5 degradation	(147)
	Deubiquitination	USP3	(K63-linked)	MDA5 inhibition	(133)
	Ubiquitination	TRIM65	hK743 (K63-linked)	MDA5 oligomerization and activation	(148)
	Dephosphorylation	PP1	hS88	MDA5 activation	(141)
	Phosphorylation	RIOK3	hS828	MDA5 inhibition by impairing multimer formation	(149)
	SUMO	TRIM38	hK43/K865	MDA5 stabilization and activation	(143)
	SUMO	SENP2		MDA5 degradation and inhibition	(143)
	ISGylation			MDA5 oligomerization and activation and is antagonized by papain-like protease of SARS-CoV-2	(150)
MAVS	Ubiquitination	TRIM31	hK10/K311/K461 (K63-linked)	MAVS oligomerization and activation	(151)
	Deubiquitination	USP18		MAVS activation by recruiting TRIM31	(152)
	Ubiquitination	TRIM21	hK325 (K27-linked)	MAVS activation to recruit TBK1	(153)
	*O-*GlcNAcylation	OGT	hS366	MAVS activation by enhancing K63-linked ubiquitination	(154)
	Deubiquitination	OTUD4	(K48-linked)	MAVS stabilization	(155)
	Ubiquitination	TRIM25	hK7/K10 (K48-linked)	MAVS degradation and release of MAVS assembled signaling complex for IRF3 activation	(156)
	Ubiquitination	YOD1	(K63-linked)	Removes K63-linked ubiquitin from MAVS and reduces MAVS aggregates	(157)
	Deubiquitination	OTUD3	(K63-linked)	MAVS inhibition	(158)
	Ubiquitination	AIP4	hK371/K420 (K48-linked)	Bridged by PCBP1/PCPB2 to target MAVS for degradation	(159, 160)
	Ubiquitination	RNF115	hK500 (K48-linked)	MAVS degradation	(88)
	Ubiquitination	pVHL	hK420 (K48-linked)	MAVS degradation	(161)
	Ubiquitination	MARCH5	hK193/K203 and/or hK7/K500 (K48-linked)	MAVS degradation	(162) (163)
	Ubiquitination	Itch	(K48-linked)	MAVS degradation mediated by TAX1BP1	(164)
	Ubiquitination	Smurf2		MAVS degradation	(165)
	Ubiquitination	Smurf1	(K48-linked)	MAVS degradation mediated by Ndfip1	(166)
	Deubiquitination	OTUD1		MAVS degradation by stabilizing Smurf1 to promote Smurf1-mediated MAVS ubiquitination	(167)
	Ubiquitination	RNF5	hK362/K461 (K48-linked)	MAVS degradation	(168)
	Ubiquitination	STUB1		MAVS degradation mediated by RACK1 after BEFV infection	(169)
	Phosphorylation	TBK1/IKKβ	hS442	MAVS activation to recruit IRF3	(81)
	Phosphorylation	cAbl		MAVS activation	(170)
	Phosphorylation	NLK	hS121/S212/S258/S329	MAVS degradation and inhibition	(171)
	Dephosphorylation	PPM1A		MAVS dephosphorylation and inhibition	(172)
	SUMOylation		SUMO3 but not SUMO1/SUMO2	MAVS aggregation enhanced	(173)
	Desuccinylation	SIRT5	hK7	MAVS aggregation reduced upon desuccinylation	(174)

The orange color indicates activation of the indicated molecule by indicated modifications; the blue color indicates suppression of the indicated molecules by indicated modifications.

Similar to STING, MAVS undergoes S442 phosphorylation by either TBK1 or IKKβ, and this phosphorylation is essential to recruit IRF3 for its phosphorylation and nuclear translocation in inducing interferon production upon sensing cytosolic RNA (81). Through a yeast two-hybrid screen, the tyrosine kinase c-Abl binds the CARD and TM domains in MAVS to phosphorylate MAVS on Y residues, through unknown mechanisms to facilitate MAVS activation (170). In contrast, NLK-mediated MAVS-S121/S212/S258/S329 phosphorylation upon RNA viral infection promotes MAVS degradation to dampen RNA sensing ability (171). PPM1A (protein phosphatase magnesium-dependent 1A) is complexed with TBK1/IKKε and targets both MAVS and TBK1/IKKε for dephosphorylation, leading to the dissociation of the MAVS/TBK1/IKKε signaling complex and subsequently impaired RNA sensing signaling. As a result, *Ppm1a* ^−/−^ mice are resistant to RNA viral infection (172).

### Ubiquitination

Activation of RIG-I requires unanchored K63-linked ubiquitin chains in addition to RNA and ATP. Free K63-linked ubiquitin chains bind to the CARD domains in RIG-I (121), allowing for a transition from a closed inactive conformation to an open active conformation. At the early phase of RNA viral infection, activation of RIG-I requires TRIM25-mediated K63-linked polyubiquitination modification on K172, which serves as a platform to recruit downstream effector binding (122). With help from E2 enzymes UBE2D3 and UBE2N, the E3 ligase Riplet facilitates the conjugation of K63-linked ubiquitination on RIG-I to aid its activation (123). Another study reports the E3 ligase REUL governs RIG-I ubiquitination (presumably through K63 linkage) on K154/K164/K172, also promoting RIG-I-mediated RNA sensing using a yeast two-hybrid assay (125). In addition, through screening human ubiquitin-related enzyme cDNA library, expression of TRIM14 is observed to be able to stimulate IFN-β promoter reporter largely through promoting K63-linked RIG-I ubiquitination on K154/K164/K172 (126). Moreover, MEX3C adds K63-linked ubiquitin chains to RIG-I K99/K169 residues to exert a similar signaling activation function (127). An independent yeast two-hybrid screen identified RNF135 as an additional E3 ligase to promote K63-linked RIG-I ubiquitination on its C-terminal K849/K851 residues, exerting a similar function as TRIM27-mediated RIG-I-K172 ubiquitination to facilitate RIG-I activation (124). With the use of distinct approaches including microarray and DUB cDNA screen, three DUBs including CYLD (132), USP3 (133), and USP21 (134) were reported to remove K63-linked RIG-I ubiquitination to antagonize RIG-I activation. CYLD maintains low RIG-I ubiquitination at the resting state, and during viral infection, CYLD is downregulated, allowing K63-linked RIG-I ubiquitination to occur for RIG-I activation (132). Notably, at the resting state, USP3 does not bind RIG-I, and upon viral infection, an induced USP3 binding to RIG-I leads to the removal of K63-linked ubiquitin chains to restrain or terminate RIG-I signaling (133). Whether USP21 expression or interaction with RIG-I is also regulated by viral infection remains unclear.

In addition to K63-linked ubiquitination that promotes RIG-I signaling complex formation, K48-linked RIG-I ubiquitination has also been observed to control RIG-I protein stability. To this end, RNF122 was observed to co-localize with RIG-I to conjugate K48-linked ubiquitin chains to K115/K146 residues that earmark RIG-I for proteasomal degradation (128). A yeast two-hybrid assay found RNF125 as a RIG-I binding E3 ligase that conjugates K48-linked ubiquitin chains to both RIG-I and MDA5 to promote their destruction (129). Expression of both RNF122 and RNF125 is enhanced by IFN production; thus, RNF122- or RNF125-mediated RIG-I ubiquitination and degradation may serve as a negative feedback mechanism to restrain sustained innate immune activation. Siglec-G induced by RNA viral infection facilitates SHP2 and the E3 ligase c-Cbl binding to RIG-I, where c-Cbl facilitates K48-linked RIG-I-K813 ubiquitination and degradation, serving as a mechanism hijacked by RNA viruses to disable RIG-I-mediated RNA sensing (131). In contrast, the deubiquitinase USP4 is found to remove K48-linked ubiquitin chains from RIG-I, thus stabilizing RIG-I to facilitate its RNA sensing function (135). USP4 expression is attenuated upon RNA viral infection; thus, USP4-mediated RIG-I deubiquitination may serve as a negative regulatory mechanism to restrain RIG-I signaling from overactivation or sustained activation.

Moreover, TRIM25-mediated RIG-I K63-linked ubiquitination and activation can also be antagonized by the linear ubiquitin assembly complex composed of HOIL-1L/HOIP/LUBAC, where HOIL-1L/HOIP targets TRIM25 for degradation, and HOIL-1L also competes with TRIM25 to bind RIG-I (130). These two mechanisms independently lead to the suppression of RIG-I activation when sensing cytosolic RNA. Moreover, the deubiquitinase USP15 removes the ubiquitin moiety from RIG-I to stabilize TRIM25, leading to enhanced RIG-I ability in sensing cytosolic RNA to promote interferon production (136).

Like RIG-I, K63-linked ubiquitination of MDA5 by TRIM65 on K743 residue is critical for MDA5 oligomerization and subsequent activation upon RNA viral infection (148). In addition, viral infection induces USP3 interaction with MDA5 to catalyze the removal of K63-linked ubiquitin chains, thus limiting sustained activation of MDA5 signaling (133). At the later stage of viral infection, expression of either the E3 ligase RNF125 (129) or TRIM13 (147) is induced, leading to conjugation of K48-linked ubiquitin chains to MDA5 for MDA5 degradation, both of which serve as a negative feedback mechanism to terminate MDA5 signaling.

Activation of MAVS can be initiated by either K63-linked or K27-linked ubiquitination events. TRIM31 conjugates K63-linked ubiquitin moieties to MAVS-K10/K311/K461, which is necessary for MAVS oligomerization and subsequent activation (151). Interestingly, a mitochondrion-localized DUB USP18 serves as a scaffold protein to bridge TRIM31 interaction with MAVS, enhancing TIRM31-mediated K63 linkage ubiquitination of MAVS for its activation (152). Expression of TRIM21 is enhanced under viral infection, where TRIM21 catalyzes K27-linked ubiquitination of MAVS-K325, which further recruits TBK1 to transduce innate immune signaling (153), which may serve as a fine-tuning mechanism to enhance innate immunity. In addition, metabolic states also modulate anti-RNA viral infection responses. For example, OGT (*O*-linked β-*N*-acetylglucosamine (*O*-GlcNAc) transferase) adds on *O*-GlcNAc to MAVS-S366 residue, which promotes K63-linked ubiquitination of MAVS for its activation (154). Another approach to enhance MAVS activation is to stabilize MAVS proteins by removing K48-linked ubiquitin chains from MAVS by OTUD4. Upon viral infection, OTUD4 expression is induced to quickly stabilize MAVS, preparing it for timely response to infection (155). Interestingly, TRIM25 targets MAVS for ubiquitination and degradation after MAVS activation, allowing the release of MAVS assembled signaling complex including TRAF3, NEMO, and TBK1 to translocate to the cytoplasm where TBK1 phosphorylates and activates IRF3 to facilitate interferon production (156).

It seems that at resting states, the levels of MAVS-K63-linked ubiquitination remain low by OTUD3, while upon viral infection, OTUD3 is inactivated by SIRT1-mediated K129 deacetylation, allowing for the buildup of K63-linked ubiquitination of MAVS for its activation (158). MAVS K63-linked ubiquitin moiety added on MAVS for its activation can be removed by YOD1 in the later stage of viral infection to restrain MAVS from overactivation (157). At the resting state, PCBP1 bridges the E3 ligase AIP4 to ubiquitinate MAVS through a K48 linkage to target MAVS for proteasomal degradation, thus maintaining a low level of MAVS expression (159). Upon viral infection, PCBP2 expression is induced and similarly bridges AIP4 to target MAVS-K371/K420 for degradation at later stages of infection, serving as a possible negative feedback mechanism to terminate MAVS signaling (159, 160). Another mechanism to maintain a low level of MAVS under uninfected conditions is achieved by RNF115-mediated K48-linked polyubiquitination on K500 for degradation of MAVS (88).

Ubiquitination of MAVS also plays an important role in preventing sustained activation of the MAVS signaling. To this end, viral infection induces RNF5 binding to MAVS, leading to RNF5-mediated conjugation of K48-linked ubiquitin chains to MAVS-K362/K461 leading to MAVS destruction (168). Similarly, viral infection also induces binding of the mitochondrial E3 ligase MARCH5 to aggregated and active MAVS, where MARCH5 ubiquitinates K193/K203 (162) or K7/K500 (163) through a K48 linkage for MAVS destruction, serving as a negative feedback mechanism to restrain sustained MAVS activation. In addition, viral infection induces expression of TAX1BP1, which recruits the E3 ligase Itch to add on K48-linked ubiquitination to MAVS for MAVS degradation in terminating MAVS signaling (164). Another E3 ligase Smurf2 (Smad ubiquitin regulatory factor 2) also promotes MAVS ubiquitination through a K48 linkage to promote MAVS destruction (165). Similarly, Smurf1 also promotes K48-linked ubiquitination and destruction of MAVS, which depends on Ndfip1 as a recruiter and activator for Smurf1 (166). In addition, RNA viral infection induces OTUD1 expression, which deubiquitinates and stabilizes Smurf1, therefore enhancing Smurf1-mediated MAVS degradation to negatively regulate MAVS function (167). The E3 ligase pVHL also negatively controls MAVS protein stability by adding K48-linked polyubiquitin chains on MAVS-K420 to facilitate its destruction (161).

RNA viruses also hijack MAVS degradation mechanisms to facilitate viral replication and viral infection. For example, RNA viral infection enhances the expression of RACK1 (Receptors for activated C kinase 1), by upregulating the expression of the E3 ligase STUB1 (STIP1 homology and U-box containing protein 1) to target MAVS for ubiquitination and degradation; RACK1 facilitates BEFV (bovine epidemic fever virus) replication (169).

### SUMOylation and Neddylation

TIRM38 exerts a protein SUMO E3 ligase activity in governing SUMOylation of RIG-I-K96/K888 and MDA5-K43/K865 in uninfected and early infected cells, respectively, to stabilize both RIG-I and MDA5 by antagonizing K48-linked ubiquitination, leading to an acute and enhanced response to viral infection (143). At the later infection stage, SENP2 removes SUMO conjugates to facilitate RIG-I and MDA5 proteasomal degradation to terminate RNA sensing signaling (143). Recently, ISGylation of MDA5 has also been reported to facilitate MDA5 oligomerization and activation, a process that can be antagonized by the papain-like protease of SARS-CoV-2 (150). In addition, SUMO3, not SUMO2 or SUMO1, addition to MAVS has been reported to enhance MAVS aggregation and activation to stimulate interferon production (173) upon poly(dA:dT) treatments.

### Other Protein Modifications

Upon viral infection, HDAC6 binds and deacetylates RIG-I-K909 to enhance its RNA sensing ability by allowing the formation of RIG-I oligomers (145, 146). The viral PFAS (phosphoribosylformylglycinamide synthase), although lacking intrinsic activity, uses host PFAS to deamidate RIG-I on Q10/N245/N445 to activate RIG-I in triggering the host RNA sensing signaling (144). In addition, after viral infection, SIRT5 catalyzes MAVS desuccinylation at residue K7 to reduce the MAVS aggregates to limit MAVS activation and RLR signaling (174).

## Conclusions

Given that hyperactivation of cytosolic nucleic acid sensing signaling causes autoimmune diseases while hypoactivation of cytosolic nucleic acid sensing leads to susceptibility to infection and compromised immunotherapeutic effects [summarized in (31)], the timely and concise control of activation of both cytosolic DNA and RNA sensing signaling is tightly controlled through multilayer regulatory mechanisms. Among them, post-translational modifications of key cytosolic nucleic acid sensing pathway members have been extensively studied, and fine-tuning mechanisms have been elucidated. Considering the structural difficulties in distinguishing pathogen DNA or RNA from the host’s nucleic acids, the innate immune system may prefer to enhance sensitivity during infection because the likelihood of a positive is high, and false-negative risk is acceptable for a short period of time.

In echoing this concept, post-translational modifications on key cytosolic nucleic acid sensors have been shown to differently govern sensor activation in different stages of infection. For example, cGAS is found to be unphosphorylated at the resting state to restrain its inappropriate activation by associating with PPP6C, and this interaction is alleviated upon viral infection, allowing for cGAS phosphorylation that primes cGAS activation (45). In contrast, RIG-I and MAVS are phosphorylated at resting states and upon viral infection; removal of phosphorylation on RIG-I and MAVS by PP1α and PP1γ is required for their activation (141, 142). In addition, the expression of a handful of E3 ligases and DUBs is induced by interferons; thus, ubiquitination or deubiquitination of nucleic acid sensors serves as a fine-tuning mechanism to restrain sustained innate immune signaling or terminate nucleic acid sensing signaling. For example, interferon induces expression of TRIM21, which ubiquitinates and degrades IFI16 (59) and DDX41 (68) to restrain DNA sensing, while facilitating K27-linked MAVS (153) to facilitate RNA sensing. HSV-1 infection induces TRIM30a expression, which targets STING for ubiquitination and degradation to shut down interferon signaling (84). Similarly, interferon induces expression of RNF122 (128) and RNF125 (129) to ubiquitinate and degrade RIG-I/MDA5, triggers TRIM13 synthesis to degrade MDA5 (147), or promotes RNF5 (168) and MARCH5 (162, 163) expression to degrade MAVS, all leading to inactivation of RNA sensing signaling after viral infection. Interferon induces downregulation of CYLD to promote RIG-I K63-linked ubiquitination for its activation (132) and reduces OTUD3 expression to allow MAVS to undergo K63-linked ubiquitination and activation (158). Moreover, interferon production also interferes with USP3 binding with both RIG-I (133) and MDA5 (133), allowing for the buildup of K63-linked ubiquitin chains for their activation. Aberrant activation of resting-state nucleic acid sensors might contribute to autoimmune diseases. This can be achieved by modulating nucleic acid sensor-modifying enzymes mentioned above. To this end, PP2A has been reported to confer susceptibility to autoimmune diseases (175). In addition, connections and crosstalks between innate immune responses and tumorigenesis are also observed. For example, TRIM21 facilitates tumorigenesis through ubiquitinating tumor-suppressive (e.g., p53 and p21) substrates (176). Given TRIM21 expression is induced by interferon to terminate DNA sensing (59), whether nucleic acid sensing-related function of TRM21 also contributes to tumorigenesis by evading cytosolic DNA sensing remains to be determined.

Interestingly, K63-linked ubiquitination has been shown to play a critical role in facilitating both cytosolic DNA and RNA sensing. K63-linked ubiquitination of STING is not only important for proper STING conformational changes and proper trafficking (89, 92) but also critical for recruiting its downstream effectors TBK1 and IRF3 (88, 91). Similarly, un-anchored K63-linked ubiquitin chains are necessary to activate RIG-I (121), and K63-linked ubiquitination of RIG-I by TRIM25 is also pivotal for RIG-I aggregate formation and activation (122). Conjugating K63-linked ubiquitin chains to MDA5 by TRIM65 is critical for MDA5 oligomerization and activation (148). MAVS ubiquitination by TIRM31 *via* a K63 linkage is required for its activation (152). To this end, K27-linked ubiquitination of cGAS has been reported to facilitate cGAS activation (47), while whether K63-linked ubiquitination plays a similar activation function remains to be further determined. In cancer, K63-linked ubiquitination has been observed to promote activation of oncogenic kinases including Akt and TAK1 (177) and various DNA damage response factors including CLASPIN (178), both leading to enhanced tumorigenesis.

It is commonly observed that a given protein can be modified by multiple posttranslational modifications to control distinct functions in a temporal and spatial manner (31). One residue in a given protein can also be regulated by different modifications. For example, TRIM56 mono-ubiquitinates mcGAS on K335 to facilitate its activation (46), while SENP7 deSUMOylates mcGAS-K335 (46). This may suggest that these modifications are mutually exclusive, and it is plausible that deSUMOylation is necessary for cGAS mono-ubiquitination for cGAS activation. In contrast, the same enzyme also controls multiple target functions. For example, TRIM38 functions as a SUMO ligase to differentially control cytosolic nucleic acid sensing. Specifically, TRIM38 SUMOylates cGAS (51) and STING (51) to stabilize both of them by antagonizing degradation-oriented ubiquitination, thus facilitating activation of DNA sensing. Similarly, TRIM38 also SUMOylates RIG-I (143) and MDA5 (143) to stabilize RIG-I and MDA5 for activation. Thus, distinct nucleic acid sensor-modifying enzymes can coordinate or compete by competitively regulating the same residues.

**Figure 3:**
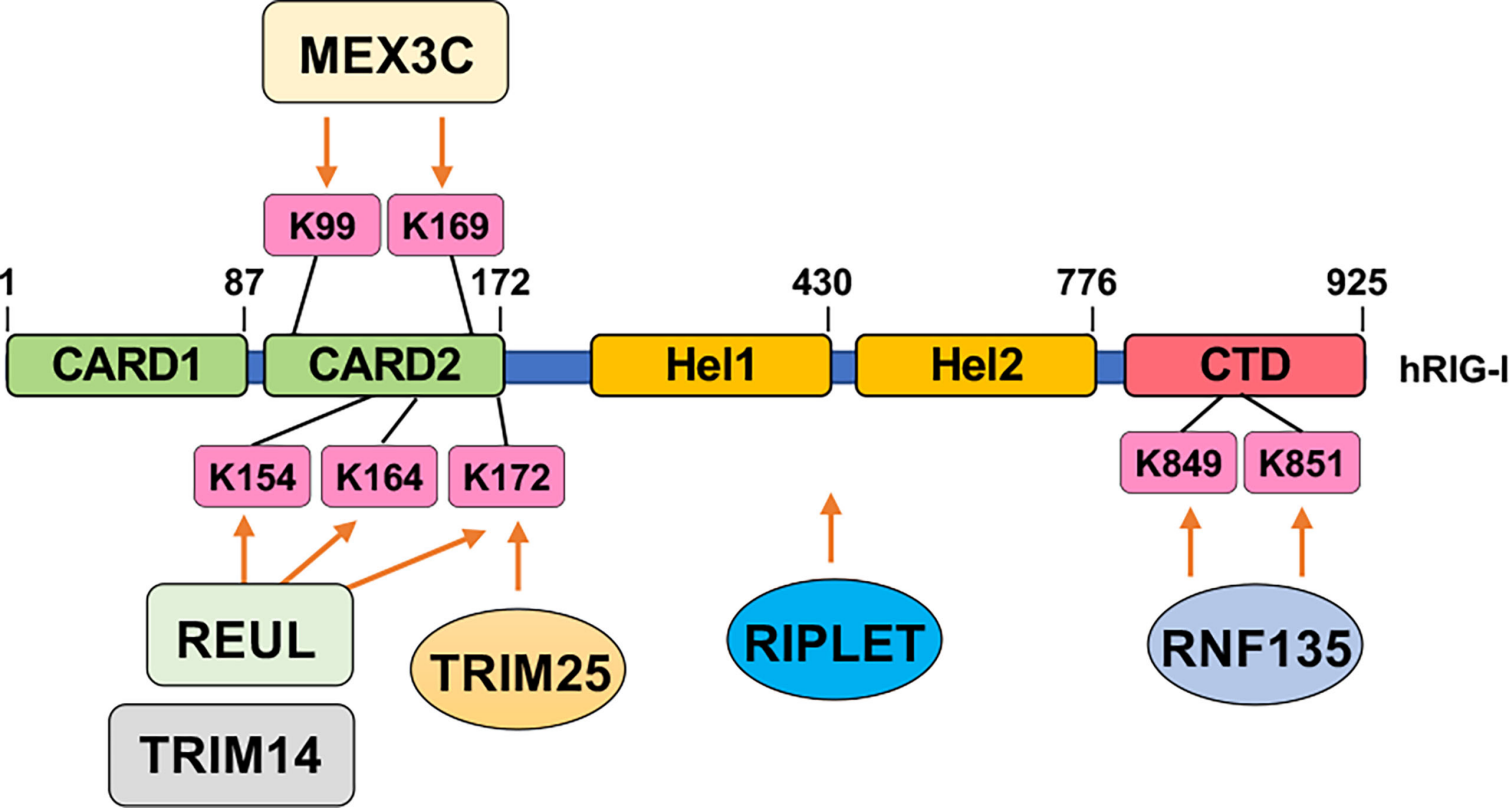
Ubiquitination-mediated RIG-I activation. Indicated E3 ubiquitin ligases add on K63-linked ubiquitin chains to indicated residues in RIG-I to facilitate RIG-I activation upon sensing cytosolic RNA.

In addition to the timing of the modification (e.g., prior to or post-infection), the location of the modifications most of the time also dictates distinct functions. For example, three DNA binding sites termed site A, site B, and site C are identified in cGAS (179), which span the whole cGAS molecule. Acetylation of the cGAS-N terminus (K47/K56/K62/K83) facilitates DNA binding (55), while deacetylation of the cGAS C-terminus (K384/K394/K414) enhances cGAS binding with DNA (56). Another example is for fine-tuned activation control of RIG-I by multiple ubiquitination events (**Figure 3**). Specifically, TRIM25-mediated K172 ubiquitination is necessary for RIG-I activation (122). Riplet introduces K63-linked ubiquitination to activate RIG-I (123). Similarly, another two E3 ligases including REUL (125) and TRIM4 (126) add on K63-linked ubiquitin conjugates to K154/K164/K172 for RIG-I activation. In addition, MEX3C aids K63-linked ubiquitination on K99/K169, which also promotes RIG-I activation (127). Even K63-linked ubiquitination on C-terminal K849/K851 by RNF135 facilitates RIG-I activation (124). Why there is a need for multiple E3 ligases to catalyze the same ubiquitination events, why ubiquitination at distinct residues all lead to RIG-I activation, and whether these modifications/expression of enzymes are viral type, infection stage, or tissue-specific remain to be further determined. Nonetheless, these examples reveal multilayers of regulatory mechanisms achieved by the same or crosstalks among different posttranslational modifications. Moreover, whether these modifying enzymes can be targeted for treating patients with either autoimmune diseases or immune deficiency warrants a promising yet understudied direction.

Among all the nucleic acid-modifying enzymes, inhibitors targeting kinases have been developed for the treatment of autoimmunity and inflammation, including JAK, IRAK4, RIPK, SYK, BTK, and TPL2 (180). Targeting kinases regulating nucleic acid sensor activities like Akt is also feasible—Akt inhibition suppresses tumor growth not only through intrinsic survival mechanisms but also through releasing Akt-mediated cGAS suppression to facilitate innate immune activation and subsequent increased immune cell infiltrates, which warrants further investigations. In contrast, the application of BLK inhibitors in treating T-cell lymphoma (where BLK was shown as an oncogene (181)) should be used with caution because inhibiting BLK may attenuate cGAS cytosolic retention leading to deficiency in cGAS activation and dampened T-cell recruitment into tumors. In contrast, inhibitors targeting E3 ligases and DUBs are not well developed largely because E3 ligases do not exert enzymatic activity and technical difficulties in developing DUB inhibitors. However, it is plausible that properly targeting nucleic acid sensor-modifying enzymes listed in this review will lead to new therapeutic directions for treating either autoimmune diseases/inflammation or cancer.

## Author Contributions

YD and YW: information collection. YD and YW: table/figure construction. YD, YW, and PL: drafting of the manuscript. YD, YW, LL, EM, and PL: revising of the manuscript. All authors listed have made a substantial, direct, and intellectual contribution to the work and approved it for publication.

## Funding

This work was supported by an NIH grant (R01CA244825, PL), the Gabrielle’s Angel Foundation Medical Research Award (PL), and the UNC University Cancer Research Fund (PL).

## Conflict of Interests

The authors declare that the research was conducted in the absence of any commercial or financial relationships that could be construed as a potential conflict of interest.

## Publisher’s Note

All claims expressed in this article are solely those of the authors and do not necessarily represent those of their affiliated organizations, or those of the publisher, the editors and the reviewers. Any product that may be evaluated in this article, or claim that may be made by its manufacturer, is not guaranteed or endorsed by the publisher.
